# Immunogenicity of a Fractional Dose of mRNA BNT162b2 COVID-19 Vaccine for Primary Series and Booster Vaccination among Healthy Adolescents

**DOI:** 10.3390/vaccines10101646

**Published:** 2022-09-30

**Authors:** Thanyawee Puthanakit, Napaporn Chantasrisawad, Kirana Yoohat, Rapisa Nantanee, Jiratchaya Sophonphan, Thutsanun Meepuksom, Pimpayao Sodsai, Supranee Phanthanawiboon, Watsamon Jantarabenjakul, Nattiya Hirankarn, Pope Kosalaraksa

**Affiliations:** 1 Division of Infectious Diseases, Department of Pediatrics, Faculty of Medicine, Chulalongkorn University, Bangkok 10330, Thailand; 2 Center of Excellence for Pediatric Infectious Diseases and Vaccines, Faculty of Medicine, Chulalongkorn University, Bangkok 10330, Thailand; 3 Thai Red Cross Emerging Infectious Diseases Clinical Center, King Chulalongkorn Memorial Hospital, Bangkok 10330, Thailand; 4 Monoclonal Antibody Production and Application Research Team, National Center for Genetic Engineering and Biotechnology (BIOTEC), National Science and Technology Development Agency (NSTDA), Pathum Thani 12120, Thailand; 5 Center of Excellence for Allergy and Clinical Immunology, Division of Allergy, Immunology and Rheumatology, Department of Pediatrics, Faculty of Medicine, Chulalongkorn University, Bangkok 10330, Thailand; 6 Center of Excellence in Immunology and Immune-Mediated Diseases, Department of Microbiology, Faculty of Medicine, Chulalongkorn University, Bangkok 10330, Thailand; 7 Department of Microbiology, Faculty of Medicine, Khon Kaen University, Khon Kaen 40002, Thailand; 8 Division of Infectious Diseases, Department of Pediatrics, Faculty of Medicine, Khon Kaen University, Khon Kaen 40002, Thailand

**Keywords:** SARS-CoV-2 vaccine, adolescents, neutralizing antibody titer, anti-SARS-CoV-2, fractional dose, BNT162b2, booster dose

## Abstract

Primary series vaccination with BNT162b2 followed by a booster 5 months later has been recommended for healthy adolescents. We aimed to describe the immunogenicity in a fractional dose of BNT162b2. Adolescents aged 12–18 years were randomized into six arms for primary series administration: 3wPZ30/30 (reference group), 3wPZ30/20, 3wPZ20/20, 6wPZ30/30, 6wPZ30/20, and 6wPZ20/20 μg. A booster was given at 5 months after the second dose using either 10 or 15 μg of BNT162b2. Immunogenicity following vaccination was determined by IgG against receptor-binding domain (anti-S-RBD IgG; BAU/mL), surrogate virus neutralization test (sVNT; %inhibition) and pseudovirus neutralization (pVNT;ID_50_) against Omicron. Non-inferiority criteria were defined as a lower boundary of the geometric mean ratio (GMR) being greater than 0.67. From September to October 2021, 118 adolescents with a median age (IQR) of 14.9 years (13.9–16.7) were enrolled. Fourteen days after the primary series, the geometric means (GMs) of anti-S-RBD IgG (BAU/mL) were 3090 (95% CI 2761–3460) in 3wPZ30/30. The GMRs of anti-S-RBD were: 0.80 (95% CI 0.67–0.97) in 3wPZ30/20; 1.00 (95% CI 0.83–1.20) in 3wPZ20/20; 1.37 (95% CI 1.13–1.65) in 6wPZ30/30; 1.24 (95% CI 1.02–1.50) in 6wPZ30/20; and 1.36 (1.13–1.64) in 6wPZ20/20. After a booster dose with 15 μg (n = 24) of BNT162b2, sVNT and pVNT against Omicron variant were 91.6 (95% CI 88.4–94.9) and 331 (95% CI 221–495), respectively. In the group that received 10 μg of BNT162b2 (n = 25), sVNT was 85.6 (95% CI 80.0–91.6) and pVNT was 397 (95% CI 267–590). Healthy adolescents had good immune responses to the fractional dose regimen of BNT162b2 and this may be considered as an alternative option.

## 1. Introduction

As of August 2022, severe acute respiratory syndrome coronavirus 2 (SARS-CoV-2) has infected around 596 million people worldwide, causing more than 6.4 million deaths [1]. In Thailand, more than 4.6 million cases of COVID-19 have been reported, with 17% or 775,000 cases being in children and adolescents [2]. The dominant circulating strain of SARS-CoV-2 in Thailand has changed over time, with the Delta variant (B.1.617.2) circulating during May to December 2021 [3], then the Omicron variant (B.1.1.529) in January 2022, which reached almost 100% in March 2022 [4]. Preventive measures against COVID-19 in children, similar to adults, consists of wearing masks, regular handwashing, and social distancing, in combination with vaccination against SARS-CoV-2 [5]. BNT162b2 COVID-19 vaccine, manufactured by Pfizer-BioNTech, contains a nucleoside-modified messenger RNA encoding the SARS-CoV-2 spike glycoprotein [6,7] and received authorization for emergency use in adolescents aged 12–18 using a 30 μg dose, the same as in adults [8]. In October 2021, the Thai Ministry of Public Health recommended two doses of BNT162b2 for healthy adolescents. Since 2022, Omicron has been the dominant variant circulating globally. Data from PROTECT cohort in the U.S. show that vaccine effectiveness during the Omicron predominant period is only 59%, significantly lower than during the Delta predominant period where effectiveness was 87% [9]. In April 2022, Thailand recommended a booster dose of BNT162b2 for adolescents aged 12–18 years with an interval of at least 4–6 months following the second dose.

Myocarditis has been reported as an adverse effect of BNT162b2 and is of special interest due to the vaccine hesitancy it created, reported in males more than females. Myocarditis incidence is 70.7 cases per million doses among male adolescents aged 12–15 years old—higher in the second dose than the first dose [10]. BNT162b2 vaccine received emergency use authorization for children aged 5 to 11 years old using one-third the dose that adolescents received (10 μg) [11]. Therefore, a fraction of the BNT162b2 adult dose (30 μg) among adolescents as primary series administration (e.g., 20 μg) should be explored. The CoV-Boost study, a prime-boost strategy among healthy adults in the U.K., found that a half-dose of BNT162b2 provided a high immune response [12]. An alternative vaccine, mRNA-1273, was authorized by the U.S. FDA to be given as a booster dose in October 2021 as a 50 μg dose, a half dose [13]. In addition, in resource-limited settings where access to vaccines is limited, a fractional lower dose approach may increase vaccine coverage at the population level.

Therefore, this study aims to describe the immunogenicity of the fractional lower dose of BNT162b2 as a primary series vaccination and a booster dose among healthy adolescents.

## 2. Materials and Methods

### 2.1. Study Design and Participants

This study is a double-blinded, randomized clinical trial registered as Thai Clinical Trial number TCTR20210917004. Inclusion criteria were (1) healthy adolescents aged 12 to 18 years and (2) no history of COVID-19 vaccination. Exclusion criteria were participants whom: (1) had previous SARS-CoV-2 infection by medical history or positive anti-nucleocapsid test; (2) have a history of anaphylaxis to any of the BNT162b2 vaccine components; (3) received immunoglobulins or blood products 3 months prior to the first vaccination; (4) received inactivated vaccines 14 days or live vaccines 28 days prior to enrollment; (5) received immunosuppressive drugs; (6) have any acute illness within 14 days before the first vaccination. This study was conducted at two clinical research sites; the Center of Excellence for Pediatric Infectious Diseases and Vaccines, Faculty of Medicine, Chulalongkorn University and the Department of Pediatrics, Faculty of Medicine, Khon Kaen University. The institutional review board of the Faculty of Medicine, Chulalongkorn University, (IRB no 719/64) and Khon Kaen University (IRB no HE641454) approved the protocol. Informed consent was obtained from parent(s) and assent from participants prior to conducting the study procedure. This study was funded by National Vaccine Institute, Thailand (grant number 2564.1/6).

### 2.2. Study Procedure

Participants were randomly assigned into 6 arms with each arm consisting of 20 participants. The participants were vaccinated with a primary series of 2 doses of either BNT162b2 Pfizer-BioNTech lot number 30125BA 0.3 mL (30 μg, standard dose) or 0.2 mL (20 μg, fractional dose). Vaccines were given intramuscularly at the deltoid region with an interval between the first and second dose being 3 or 6 weeks. One arm received a standard dose of 30 μg with a 3-week interval (3wPZ30/30); one arm received a standard dose of 30 μg followed by a 20 μg dose 3 weeks later (3wPZ30/20); one arm received a fractional lower dose of 20 μg followed by another 20 μg dose 3 weeks later (3wPZ20/20); one arm received a standard dose of 30 μg followed by another 30 μg dose 6 weeks later (6wPZ30/30); one arm received a 30 μg dose followed by a 20 μg dose 6 weeks later (6wPZ30/20); and one arm received two 20 μg doses with a 6-week interval (6wPZ20/20). Five milliliters of venous blood samples were drawn to assess humoral immunogenicity at baseline, prior to administration of the second dose (at 3 or 6 weeks after the first dose depending on the randomization arms), at 14 days post the second dose and at 5 months post the second dose.

Cell-mediated immune responses (CMI) were assessed in 10 participants per group, with a total of 60 participants (CMI sub study). Additional 8 mL blood samples were collected at 14 days and 5 months after the second dose for evaluation of T cell responses by enzyme-linked immunospot (ELISpot) assay. SARS-CoV-2 specific memory B cell responses were measured as a single timepoint at 5 months after the second dose of BNT162b2.

The solicited local and systemic reactogenicity such as pain at injection site, fever, fatigue, myalgia, and headache within 7 days following each vaccination were recorded in a diary. The level of reactogenicity was graded into 4 levels. Grade 0: no symptoms, grade 1: mild symptoms that did not interfere with activity or a fever with body temperature (BT) 38.0–38.4 °C, grade 2: moderate symptoms with some interference with activity or a fever with BT 38.5–38.9 °C, and grade 3: severe symptoms which significantly limited daily activity or a fever with BT 39.0–40.0 °C [14].

During March 2022, a time where the Omicron variant predominantly circulated, we amend the protocol to study immune responses of a booster dose. Participants were randomized into 2 arms with one receiving BNT162b2 as a fractional lower dose of 15 μg (half adult dose, 0.15 mL, lot number PCA0062) and one fractional lower dose of 10 μg using the pediatric BNT162b2 formulation (0.2 mL, lot number FN4074). Participants were followed up at 14 days following booster for immunogenicity against the Omicron variant and at 6 months after the booster dose.

### 2.3. Immunogenicity Outcomes

Anti-nucleocapsid antibody was assessed at baseline to exclude participants who had previous infection from immunogenicity cohort of the study. Humoral immune response to vaccination was measured using 3 methods: IgG against spike protein receptor-binding domain (anti-S-RBD IgG); surrogate virus neutralization test (sVNT) against SARS-CoV-2 Delta or Omicron (BA.1) strains; and pseudovirus neutralization test (pVNT) against the Omicron (BA.2) variant according to the circulating variant. The geometric means (GMs) of antibodies of each arm were measured and compared with the standard 3wPZ30/30 regimen with a geometric mean ratio (GMR).

#### 2.3.1. Quantitative IgG against Spike Protein Receptor Binding Domain of Ancestral Strain (Anti-S-RBD IgG) ELISA

The ELISA protocol was adapted from Amanat et al. [15] and performed as described previously [16]. Briefly, diluted serum samples (at 1:5000) were incubated in 96-well plates coated with purified recombinant Myc-His-tagged S-RBD residues 319–541 from SARS-CoV-2 (Wuhan-Hu-1). Then, ELISA was performed. HRP-tagged human IgG was used as a secondary antibody. Anti-S-RBD IgG level was measured by converting OD450 into binding-antibody units (BAU/mL) using the standard curve of known units of WHO international standard (NIBSC 20/136).

#### 2.3.2. Surrogate Virus Neutralization Test (sVNT)

The surrogate virus neutralization test was adjusted from Tan et al. [17] and performed as described previously [16], utilizing the HRP-tagged recombinant S-RBD from Delta (B.1.617.2) and Omicron (B.1.1.529; BA.1) strains. Recombinant S-RBD and the ectodomain of human ACE2 were produced and purified from Human Embryonic Kidney (HEK) 293T cells transfected with expression plasmids carrying these genes. Serum samples (at 1:10 dilution) and HRP-tagged S-RBD were incubated at 25 °C for one hour before addition into 96-well plates coated with 0.1 μg/well recombinant human ACE2 ectodomain. Then, ELISA was performed and measured with OD450. Pre-2019 human serum was used as the negative samples. The % inhibition was calculated as follows:

$$\%\;inhibition=100\times \left[1-\frac{sample\;OD450\;}{negative\;OD450}\right]$$

#### 2.3.3. Pseudovirus Neutralization Test (pVNT)

Pseudovirus neutralization test (pVNT) against the Omicron variant was performed as described previously [18]. Two-fold serial dilutions of serum samples (starting 1:40) were incubated with pseudoviruses displaying the Omicron (B.1.1.529; BA.2) spike in a 1:1 vol/vol ratio in a 96-well culture plate for 1 h at 37 °C. Subsequently, suspensions of HEK293T-ACE-2 cells (2 × 10^4^ cells/mL) were mixed with the serum–pseudovirus mixture and seeded into each well. At 48 h, the levels of neutralizing antibodies were determined based on luciferase activity following entry of pseudovirus. Values were normalized against signals from no-serum controls. The ID_50_ values were calculated by determining the half-maximal inhibitory dilution.

#### 2.3.4. Enzyme-Linked Immunospot (ELISpot) Assay to Evaluate T Cell and Memory B Cell Responses

ELISpot assay by a Human IFN-γ ELISpotPro^TM^ kit (Mabtech, Stockholm, Sweden) was used to assess T cell responses. Freshly isolated peripheral blood mononuclear cells (PBMCs) with 250,000 cells per well were activated with 2 μg/mL of an overlapping peptide pool from 100 peptides of SARS-CoV-2 spike (S)-defined peptides (Mabtech, Stockholm, Sweden) for 20 h. Negative control and positive control and anti-CD3 were also included. The spots were quantified with ImmunoSpot analyzer. To evaluate positive S peptide-specific responses, spot counts of negative control wells were subtracted from S peptide-stimulated wells and these spot counts are reported as spot forming unit (SFU) per million PBMCs.

For B cells, Human IgG SARS-CoV-2 RBD ELISpot PLUS (ALP) kit (Mabtech, Stockholm, Sweden) was used for evaluation SARS-CoV-2-specific memory B cell responses. Briefly, the memory B cells were differentiated into antibody-secreting cells by pre-stimulating the fresh PBMCs with R848 and IL-2 for 72 h. Unstimulated wells were also used as a negative control. Stimulated and unstimulated PBMCs (5 × 10^5^ cells per well) were added into capture anti-IgG antibody-coated plates for ELISpot and incubated for 18 h. An RBD-WASP antigen was added into RBD-specific IgG-detected well for label and MT78/145-biotinylated antibodies were added into positive control (total IgG detected well). Anti-WASP-ALP was added into RBD-specific IgG-detected well and negative control well while streptavidin-ALP was added into positive control (total IgG detected well). Spot counting was performed using the same method as T cell assay.

### 2.4. Statistical Analysis

The sample size was calculated using a non-inferiority criterion for the GMR of anti-S-RBD IgG at day 14 after complete primary series of 3wPZ30/30, 3wPZ30/20, 3wPZ20/20, 6wPZ30/30, 6wPZ30/20, and 6wPZ20/20 μg with 3wPZ30/30 as the reference arm. A minimum of 17 participants per group was required following calculations using an assumption of a 0.67 non-inferiority margin; 80% power; 0.20 geometric standard deviation; 0.95 GMR; one-sided statistical testing with 5% significance level; and a ratio of 1:1. Additionally, by accounting for potentially missing data the sample size increased by 20% yielding a total of 20 participants per group. Participants were assigned by block randomization.

Descriptive analysis was used for baseline and clinical characteristics. Continuous variables were expressed as a median (interquartile range: IQR) and categorical variables were expressed as numbers with frequencies and percentages. GMs and GMRs with 95% CI of anti-S-RBD IgG, sVNT, and pVNT were calculated by two independent sample t-tests. Non-inferiority was concluded if the lower bound of the 95% CI did not exceed 0.67.

The anti-S-RBD IgG level above 506 BAU/mL was used in this study as a lower limit for a protective antibody level, according to the previously reported association with 80% vaccine efficacy against primary symptomatic COVID-19 [19]. The World Health Organization recommend that 68% inhibition sVNT is a threshold of high titer of antibody for the ancestral, Delta, and Omicron strains during their predominant eras. Moreover, we used pVNT ID_50_ of 185 as a cut off which correlated with 80% vaccine efficacy [19].

Statistical significance was defined as *p*-value < 0.05. Stata version 15.1 (Stata Corp., College Station, TX, USA) was used for analysis.

## 3. Results

### 3.1. Study Populations

**Figure 1:**
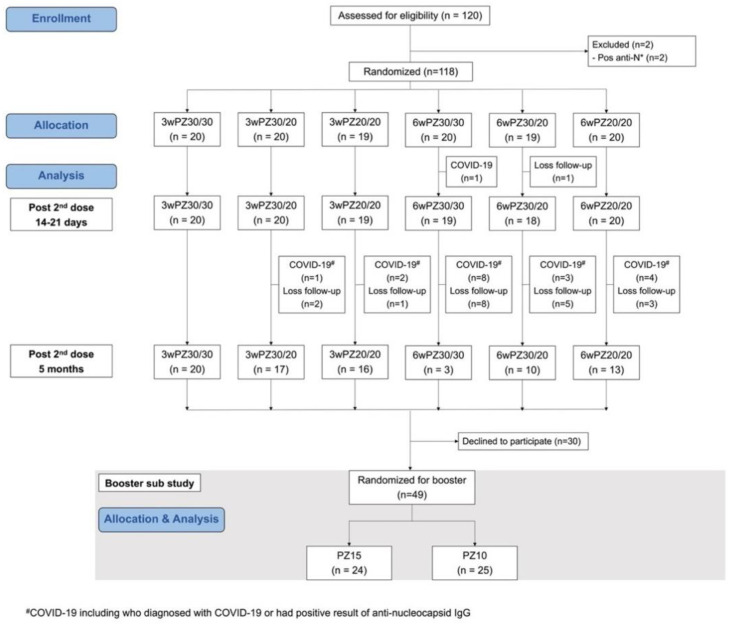
Flow diagram of study participants. * Pos anti-N: positive anti-nucleocapsid IgG; 3wPZ30/30: 3-week interval of BNT162b2 30 μg/30 μg; 3wPZ30/20: 3-week interval of BNT162b2 30 μg/20 μg; 3wPZ20/20: 3-week interval of BNT162b2 20 μg/20 μg; 6wPZ30/30: 6-week interval of BNT162b2 30 μg/30 μg; 6wPZ30/20: 6-week interval of BNT162b2 30 μg/20 μg; 6wPZ20/20: 6-week interval of BNT162b2 20 μg/20 μg; PZ15: booster with BNT162b2 15 μg; PZ10: booster with BNT162b2 10 μg.

From September to October 2021, 120 healthy adolescents were assessed for eligibility. Two of these were positive for anti-nucleocapsid antibodies and, thus, met the exclusion criteria. Therefore, 118 participants were included and randomly assigned into the six groups: 3wPZ30/30 (n = 20), 3wPZ30/20 (n = 20), 3wPZ20/20 (n = 19), 6wPZ30/30 (n = 20), 6wPZ30/20 (n = 19), and 6wPZ20/20 (n = 20), as shown in Figure 1 and Table S1. During the Delta predominant period, one participant contracted symptomatic COVID-19 (October 2021) and one participant was lost to follow-up. Therefore, 116 participants had data 14 days after completion of the two-dose primary series. During the Omicron predominant period, January to March 2022, two participants contracted symptomatic COVID-19 and 16 participants were diagnosed with asymptomatic COVID-19 by positive anti-nucleocapsid antibody. In addition, there were 19 participants lost to follow-up. A total of 79 participants had immunogenicity analysis 5 months following two-dose primary series administration. Subsequently, 49 participants consented to participate in the booster sub-study, a fractional low dose of BNT162b2, as shown in Figure 1. At 14 days after booster, 49 participants participated. At 6 months following a booster dose, 30 (61%) participants had immunogenicity analysis, of which 20 participants had SARS-CoV-2 infection (16 mild-symptom COVID-19 cases and 4 asymptomatic infections) at a median (IQR) of 114 (100–143) days post-booster dose.

### 3.2. Reactogenicity

**Figure 2:**
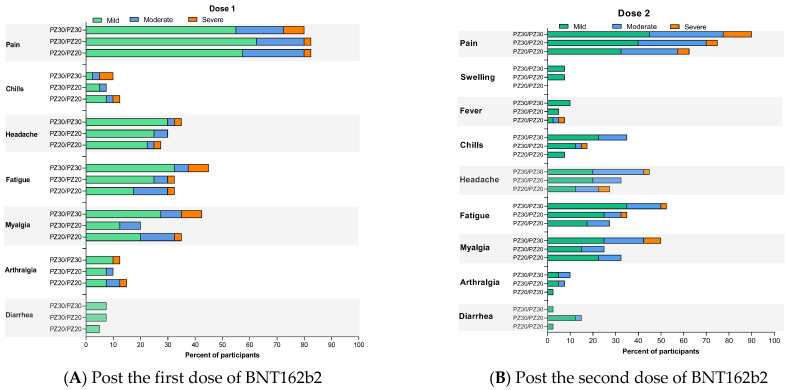
Solicited reactogenicity during 7 days after BNT162b2 primary series vaccination in healthy adolescents, by vaccination groups: (**A**) post the first dose of BNT162b2 and (**B**) post the second dose of BNT162b2.

Systemic and local reactogenicities within 7 days following vaccination were reported and shown in Figure 2 and Table S2. The most frequently reported reactogenicity was pain at injection site reported in 80–84.6% of participants after the first dose. The most common systemic reactogenicities after the first dose were fatigue reported in 39.2% (31/79) of participants who received standard dose and 33% (13/39) of participants who received fractional doses. Myalgia and headache were reported in 31.6% (25/79) and 32.9% (26/79) of the standard dose participants and 35.9% (14/39) and 28.2% (11/39) of the fractional dose participants. After the second dose, the fractional dose groups tend to have lower reactogenicities than the standard regimen. Pain at injection site was reported at a higher rate among participants who received a standard 30 μg dose; 92.3% (mild 50%, moderate 36.1%, and severe 13.9%), compared with a 20 μg dose; 70.5% (mild 52.7%, moderate 40%, and severe 7.3%). The three most common systemic reactogenicities were fatigue, myalgia, and headache that were reported in 53.8%, 51.3%, and 46.0% of the standard regimen group, respectively, whereas in the fractional-dose participants, these were reported at a lower rate; 32.0%, 29.5%, and 28.2%. No serious adverse event was reported.

Rates of common reactions after booster vaccination were similar to the rates after the primary series vaccination with a low-dose regimen. These included pain at injection site (73.5%), fatigue (44.9%), myalgia (32.7%), and headache (28.6%). Pain at injection site and headache were more commonly reported in those who received a booster with 15 μg compared to 10 μg, which were 83.3% compared to 64.0% and 41.7% compared to 16%, respectively, whereas participants who received a booster of 10 μg reported fatigue more than those who received 15 μg, which were 60.0% and 29.2%, respectively.

### 3.3. Immunogenicity

#### 3.3.1. Quantitative IgG against Spike Protein Receptor Binding Domain of Ancestral Strain (Anti-S-RBD IgG) ELISA

**Figure 3:**
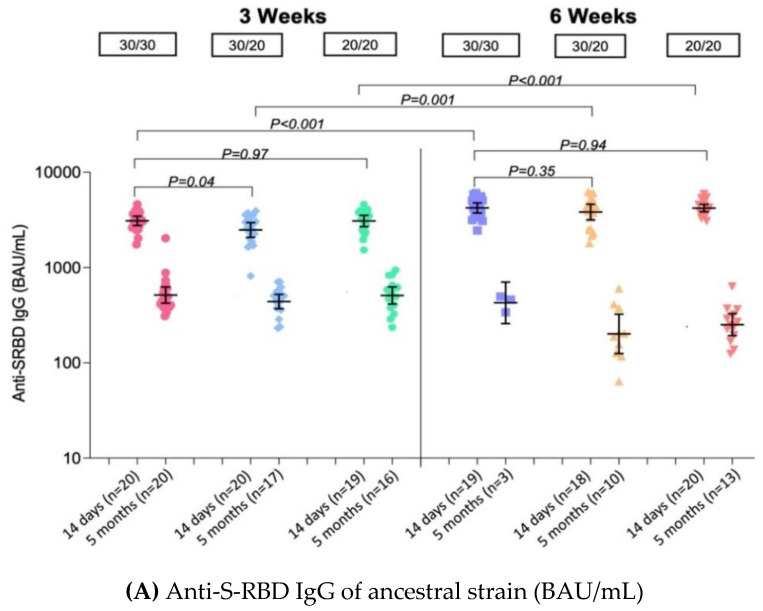

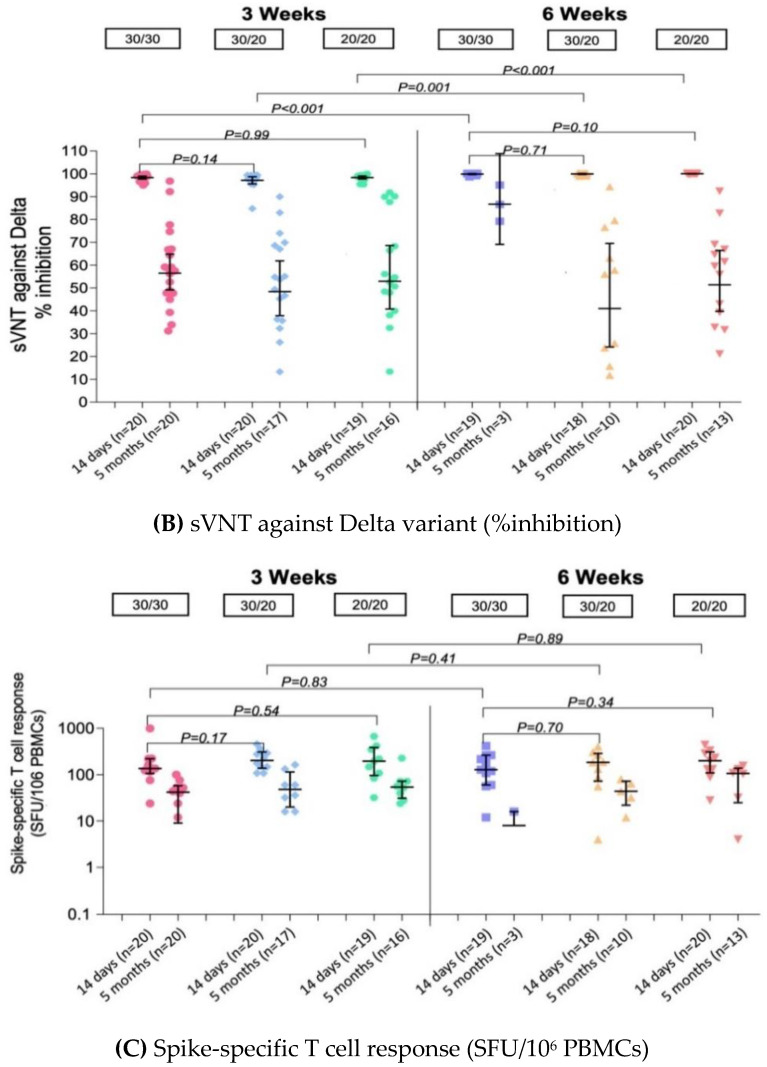
Immune response after BNT162b2 primary series vaccination in healthy adolescents by vaccination groups: (**A**) Anti-S-RBD IgG of ancestral strain (BAU/mL) (**B**) sVNT against Delta variant (%inhibition) (**C**) Spike-specific T cell response (SFU/10^6^ PBMCs) Anti-S-RBD IgG: anti spike protein receptor-binding-domain IgG; sVNT: surrogate virus neutralization test; SFU: Spot forming unit; PBMC: Peripheral blood mononuclear cell.

The GMs of anti-S-RBD IgG are shown in Table 1 and Figure 3A. After completion of the primary series vaccination, the GMs of anti-RBD IgG of SARS-CoV-2 ancestral strain were 3090 BAU/mL (95% CI 2761–3460) among adolescents who received the standard regimen of BNT162b2, 3wPZ30/30. In the fractional dose groups with a 3-week interval, the GMs were 2480 BAU/mL (95% CI 2078–2961) and 3080 BAU/mL (2685–3535) among 3wPZ30/20 and 3wPZ20/20 groups, respectively. The GMRs were 0.80 (95% CI 0.67–0.97) and 1.00 (95% CI 0.83–1.20) when compared to the standard regimen. The GMs of anti-S-RBD IgG were significantly higher among extended 6-week-interval groups, which had a GMR of 1.37 (95% CI 1.13–1.65) in 6wPZ30/30, 1.24 (95% CI 1.02–1.50) in 6wPZ30/20, and 1.36 (1.13–1.64) in 6wPZ20/20. There is no difference in the GMs of anti-S-RBD IgG across genders (*p*-value 0.94).

At the 5-month visit following the second dose of BNT162b2 at a median (IQR) of 161 days (154–161), 79 participants attended the clinic and performed the test. The GMs of anti-S-RBD IgG of the standard regimen declined to 514 BAU/mL (95% CI 423–626). The GMs of the fractional dose groups declined to 440 BAU/mL (95% CI 370–523) in 3wPZ30/20 and 509 BAU/mL (95% CI 414–627) in 3wPZ20/20, whereas, among the extended-interval groups, the GMs of anti-S-RBD IgG declined to 201–426 BAU/mL.

**Figure 4:**
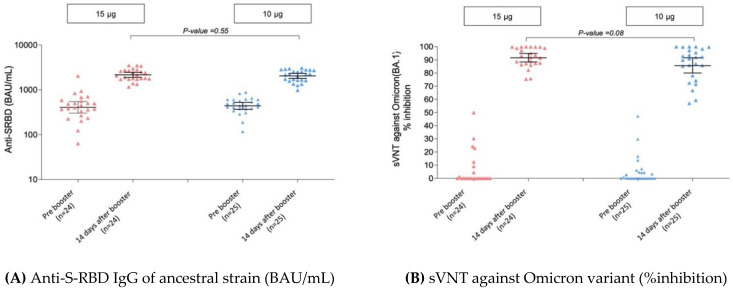
Humoral immune response after BNT162b2 booster in healthy adolescents by booster groups: (**A**) Anti-S-RBD IgG of ancestral strain (BAU/mL); (**B**) sVNT against Omicron variant (%inhibition).

Among 49 participants who received a booster dose of BNT162b2, the GMs of anti-S-RBD IgG increased by 5.3-fold in participants who received a booster of 15 μg of BNT162b2 from 407 BAU/mL (95% CI 304–546) to 2158 BAU/mL (95% CI 1897–2455) and 4.7-fold in participants who received a booster of 10 μg of BNT162b2 from 440 BAU/mL (95% CI 368–526) to 2047 BAU/mL (95% CI 1798–2330) (Table 2 and Figure 4A).

#### 3.3.2. Surrogate Virus Neutralization Test (sVNT)

The sVNT against the Delta variant of SARS-CoV-2 was assessed in the primary series vaccination cohort according to the circulating variant during the study. Data are shown in Table 1 and Figure 3B. The GM of sVNT after completing the primary series vaccination against the Delta variant among the standard regimen group (3wPZ30/30) was 98.4% inhibition (95% CI 97.7–99.0). Among the fractional-dose groups with a 3-week interval, the GMs of sVNT against the Delta variant were 97.2%inh (95% CI 95.6–98.7) and 98.4%inh (95% CI 97.7–99.0), among 3wPZ30/20 and 3wPZ20/20 groups, respectively. The GMs of sVNT against the Delta variant of the extended 6-week interval was 99.9%inh (95% CI 99.7–100.1) in 6wPZ30/30, 100%inh (95% CI 99.7–100.2) of 6wPZ30/20 group, and 100.1%inh of 6wPZ20/20 group. There is no difference in the GMs of sVNT against the Delta variant across genders (*p*-value 0.60).

At the 5-month visit after the second dose of BNT162b2, the GMs of sVNT against the Delta variant of the standard regimen declined to 56.5%inh (95% CI 49.2–64.9). The GMs of sVNT against the Delta variant among the fractional-dose groups declined to 48.4%inh (95% CI 37.9–61.9) in 3wPZ30/20 group and 52.9%inh (95% CI 40.8–68.6) in 3wPZ20/20 group, whereas, among the extended interval groups, the GMs of sVNT were 41.0–86.7%inh.

Among 49 participants who received a booster dose, sVNT against the Omicron variant (BA.1) was measured at pre and 14-days post booster (Table 2 and Figure 4B). All participants who received a booster with 15 μg of BNT162b2 achieved sVNT ≥ 68%inh against the Omicron (BA.1) variant, whereas 88% (22/25) of participants who received a booster with 10 μg of BNT162b2 achieved this target (*p*-value 0.08). After booster, the GMs of sVNT increased from 12.2%inh (95% CI 4.4–33.9) to 91.6%inh (95% CI 88.4–94.9) in participants who received 15 μg of BNT162b2 and from 6.7%inh (95% CI 3.2–13.8) to 85.6%inh (95% CI 80.0–91.6) in those who received 10 μg of BNT162b2. The GMR was 0.93 (95% CI 0.87–1.02).

At the 6-month visit following the booster dose, the GMs of sVNT against the Omicron variant (BA.1) among 10 participants who did not have natural infection were 48.4%inh (95% CI 23.2–101.2) and 48.1%inh (95% CI 12.6–183.7) in participants who received a booster of 15 μg and 10 μg of BNT162b2, respectively. Among 20 participants who had COVID-19 after receiving a booster vaccination, the GMs of sVNT against the Omicron variant (BA.1) were 97.7%inh (95% CI 95.7–99.8).

#### 3.3.3. Pseudovirus Neutralization Test (pVNT)

Data of pVNT against the Omicron (BA.2) variant following booster vaccination are shown in Table 2. The GMs of pVNT (ID_50_) were 330.7 (95% CI 221.1–494.9) in participants who received 15 μg of BNT162b2 and 396.6 (95% CI 266.4–590.4) in those who received 10 μg of BNT162b2, GMR 1.20 (95% CI 0.69–2.08).

At the 6-month visit following the booster dose, the GMs of pVNT (ID_50_) among 10 participants who did not have natural infection were 129.0 (95% CI 58.4–285.3) and 108.8 (95% CI 22.2–533.9) in those who received a booster of 15 μg and 10 μg of BNT162b2, respectively. Among 20 participants who had COVID-19 after receiving a booster vaccination, the GMs of pVNT (ID_50_) were 2611.6 (95% CI 2152.0–3169.3).

#### 3.3.4. Enzyme-Linked Immunospot (ELISpot) Assay to Evaluate T Cell and Memory B Cell Responses

The spike-specific T cell response and RBD-specific memory B cell response were evaluated. Data are shown in Table S3 and Figure 3C. As such, 14–21 days after the second dose, participants induced T cell response with a median (IQR) of 128–202 SFU/10^6^ PBMCs. There was no difference between arms. At 5 months, there were small groups of participants in whom the median of T cell was varied between 8 and 106 SFU/10^6^ PBMCs. The median of memory B cells at 5 months was 0–37 SFU/10^6^ PBMCs.

## 4. Discussion

Fractional dose of BNT162b2 for a primary series among healthy adolescents mounted a non-inferior immunogenicity response compared to a standard dose with a higher immunogenicity among groups who received a second dose at a 6-week interval, as opposed to a 3-week interval. Low-dose BNT162b2 recipients experienced less frequent local and systemic reactogenicity, especially after the second dose of BNT162b2 vaccine. There is no difference in immunogenicity across genders. After a booster dose with BNT162b2, anti-S-RBD antibody increased approximately five-fold and 88–100% of participants achieved sVNT against BA.1 ≥ 68% inhibition, which supports the policy of giving a booster dose during the Omicron-predominant period.

Humoral immune responses of adolescents who received the fractional-dose regimens for primary series were non-inferior to those who received the standard regimen. The GMRs of anti-S-RBD IgG and sVNT against the Delta variant after completion of two doses of BNT162b2 were 0.8–1.0. A study by Frenck RW et al. [7] found that SARS-CoV-2 neutralizing antibodies in adolescents were higher than young adults using the same vaccination regimen of 30 μg of BNT162b2 with a GMR of 1.76. Our study revealed that adolescents might need a lower dose to achieve an equal immunogenicity and vaccine effectiveness. A study in Thailand showed that the two most serious concerns for parents influencing COVID-19 vaccine acceptance are about vaccination side effects and safety [20], which is similar to a meta-analysis study where 61% of parents were concerned about safety of vaccines [21]. Moreover, using an epidemiological model, fractional dosing has been shown to accelerate vaccination coverage by relieving vaccine resource constraints and reducing mortality [22].

Among adolescents who received BNT162b2 with the extended 6-week-interval regimen, antibody levels were higher than those who received vaccines with the standard 3-week interval. In accordance with our results, previous studies demonstrated that longer intervals between the first and second dose improved vaccine-induced immunity [23,24]. The GM titers of anti-spike protein antibodies were 10-times higher after the second dose of BNT162b2 with 6 to 13-week intervals compared with the standard 3-week interval [24]. Studies in England [24] and Canada [25] in adults found that vaccine effectiveness was higher among those who received vaccination with an extended dosing interval longer than 6 weeks, as opposed to those who received the standard 3-week interval. Furthermore, a study among adolescents and adults in Canada showed that the extended dosing interval reduced the risk of myocarditis and pericarditis from 52.1 to 9.6 cases per million doses after two doses of BNT162b2 vaccination [26].

Cell-mediated immunity against SARS-CoV-2 plays an important role in preventing severe COVID-19 [27]. The T cell response induced by BNT162b2 vaccination was demonstrated to induce cross protection against the Omicron variant [28,29]. This study demonstrated vaccine-induced spike-specific T cell response of two doses of BNT162b2 with a median of 128–202 SFU/10^6^ PBMCs 14–21 days after completing vaccination, with no difference between the standard regimen and the fractional-dose recipients. However, T cell response in this study decreased over time in contrast with previous studies that found persistence of a T cell response following BNT162b2 vaccination for at least 3–6 months [30,31].

The Omicron variant evaded existing immunity from past infection or previous vaccination [9]. The BNT162b2 vaccine effectiveness against Omicron variant in adolescents decreased rapidly, from 59.5% after the first dose to 16.6% 2 months after the second dose, and increased to 71.1% 2 months after the booster dose [32]. Booster vaccination has been recommended and authorized in adolescents at least 5 months after the primary series by the U.S. Food and Drug Administration [33]. After a booster with fractional low dose of BNT162b2, the GMs of sVNT against the Omicron (BA.1) variant of all participants who received 15 μg of BNT162b2 achieved the target level of ≥68%inh with the GMs of 91.6%inh. This result was comparable with a previous study among adolescents who received a 30 μg of BNT162b2 as a booster dose conducted in the same laboratory, for which a median of sVNT against Omicron was 94.4%inh [34]. Although the GMs of pVNT (ID_50_) against the Omicron (BA.2) variant in fractional-dose booster groups in our study were lower than a full-dose group of a previous study [34], they were still higher than 185, which was a cut-off of 80% vaccine efficacy against symptomatic infection [19]. However, antibodies diminish over time. In the Omicron-predominant period, two-thirds of participants had COVID-19 at a median of 4 months after booster dose. This finding is similar to the COSMOS study in the United States that adjusted vaccine efficacy against emergency department visits, which declined from 60% during the first 4 months after a booster dose to only 18% thereafter [35]. Vaccines designed to target the Omicron variant are being developed and evaluated in clinical trials [36,37]. In September 2022, the U.S. Advisory Committee on Immunization recommended a booster dose with bivalent, ancestor, and the Omicron strains, vaccine for adolescents and adults ≥12 years of age [38]. However, the WHO recommend using an available ancestor vaccine as a booster shot to reduce the risk of COVID-19 infection and hospitalization.

Strengths of this study include that it was a double-blinded, randomized, controlled trial, designed to describe reactogenicity and immunogenicity outcomes of two doses of BNT162b2 with fractional-dose regimens and extended-interval regimens. Secondly, neutralizing antibodies against SARS-CoV2 were measured against the variant that predominantly circulated at each period, to retrieve information that can guide clinical practice. This study had some limitations. Firstly, the sVNT against Delta strains after the primary series reached high levels of detection >90% inhibition, meaning it might have been difficult to detect differences between groups. Secondly, due to an outbreak of the Omicron variant with a high transmissibility, large numbers of participants were infected or lost to follow-up at the 5-month visit.

## 5. Conclusions

The fractional-dose regimen for primary series vaccination tends to mount a non-inferior immune response to the Delta variant and has less reactogenicity than the standard-dose regimen. Extended intervals between administration of the first and the second vaccination doses, from 3 to 6 weeks, induced higher immunogenicity. Furthermore, the booster dose following primary series of BNT162b2 elicited good immune responses to the Omicron variant. However, due to waning of the antibody and immune escape property of the Omicron variant, natural infection with mild symptoms occurred with a median time of 4 months after booster dose.

## Figures and Tables

**Table 1:** Humoral immune response after BNT162b2 primary series vaccination in healthy adolescents by vaccination groups.

	3-Week Interval			6-Week Interval		
Groups	3wPZ30/30	3wPZ30/20	3wPZ20/20	6wPZ30/30	6wPZ30/20	6wPZ20/20
Anti-S-RBD IgG of Ancestral Strain (BAU/mL)						
Pre 2nd dose, GMs (95% CI)	*n* = 20	*n* = 20	*n* = 19	*n* = 19	*n* = 19	*n* = 20
	490	503	557	303	357	239
	(399–601)	(409–618)	(445–695)	(227–406)	(241–527)	(183–313)
Post 2nd dose: 14–21 days, GMs (95% CI)	*n* = 20	*n* = 20	*n* = 19	*n* = 19	*n* = 18	*n* = 20
	3090	2480	3080	4226	3823	4205
	(2761–3460)	(2078–2961)	(2685–3535)	(3745–4769)	(3162–4622)	(3852–4590)
GMR	ref	0.80	1.00	1.37	1.24	1.36
		(0.67–0.97)	(0.83–1.20)	(1.13–1.65)	(1.02–1.50)	(1.13–1.64)
Post 2nd dose: 5 months, GMs (95% CI)	*n* = 20	*n* = 17	*n* = 16	*n* = 3	*n* = 10	*n* = 13
	514	440	509	426	201	251
	(423–626)	(370–523)	(414–627)	(259–702)	(125–324)	(193–328)
GMR	ref	0.86	0.99	0.83	0.39	0.49
		(0.64–1.14)	(0.74–1.32)	(0.48–1.42)	(0.28–0.55)	(0.36–0.67)
sVNT against Delta variant (%inhibition)						
Pre 2nd dose, GMs (95% CI)	*n* = 20	*n* = 20	*n* = 19	*n* = 19	*n* = 19	*n* = 20
	60.9	62.6	64.8	48.5	56.8	51.7
	(54.8–67.8)	(56.1–69.8)	(55.8–75.2)	(40.7–57.9)	(47.9–67.3)	(46.3–57.6)
Post 2nd dose: 14–21 days, GMs (95% CI)	*n* = 20	*n* = 20	*n* = 19	*n* = 19	*n* = 18	*n* = 20
	98.4	97.2	98.4	99.9	100.0	100.1
	(97.7–99.0)	(95.6–98.7)	(97.7–99.0)	(99.7–100.1)	(99.7–100.2)	(100.0–100.1)
GMR	ref	0.99	1.00	1.02	1.02	1.02
		(0.98–1.00)	(0.99–1.01)	(1.00–1.03)	(1.01–1.03)	(1.01–1.03)
Post 2nd dose: 5 months, GMs (95% CI)	*n* = 20	*n* = 17	*n* = 16	*n* = 3	*n* = 10	*n* = 13
	56.5	48.4	52.9	86.7	41.0	51.4
	(49.2–64.9)	(37.9–61.9)	(40.8–68.6)	(69.1–108.9)	(24.2–69.5)	(39.7–66.5)
GMR	ref	0.86	0.94	1.54	0.73	0.91
		(0.63–1.17)	(0.69–1.28)	(0.86–2.74)	(0.51–1.04)	(0.65–1.27)

Anti-S-RBD IgG: anti spike protein receptor-binding-domain IgG; sVNT: surrogate virus neutralization test; GMs: geometric means; GMR: geometric means ratio; 95% CI: 95% confidence interval; 3wPZ30/30: 3-week interval of BNT162b2 30 μg/30 μg; 3wPZ30/20: 3-week interval of BNT162b2 30 μg/20 μg; 3wPZ20/20: 3-week interval of BNT162b2 20 μg/20 μg; 6wPZ30/30: 6-week interval of BNT162b2 30 μg/30 μg; 6wPZ30/20: 6-week interval of BNT162b2 30 μg/20 μg; 6wPZ20/20: 6-week interval of BNT162b2 20 μg/20 μg.

**Table 2:** Humoral immune response after BNT162b2 booster in healthy adolescents by booster groups.

	Booster 15 μg (*n* = 24)	Booster 10 μg (*n* = 25)	*p*-Value
sVNT against Omicron (BA.1) variant (%inhibition)			
Pre booster dose, GMs (95% CI)	12.2	6.7	0.28
	(4.4–33.9)	(3.2–13.8)	
Post booster dose: 14 days, GMs (95% CI)	91.6	85.6	0.08
	(88.4–94.9)	(80.0–91.6)	
GMR	ref	0.93 (0.87–1.01)	
pVNT against Omicron (BA.2) variant (ID_50_), GMs (95% CI)			
Post booster dose: 14 days, GMs (95% CI)	330.7	396.6	0.51
	(221.1–494.9)	(266.4–590.4)	
GMR	ref	1.20 (0.69–2.08)	
Anti-S-RBD IgG of ancestral strain (BAU/mL)			
Pre booster dose, GMs (95% CI)	407	440	0.64
	(304–546)	(368–526)	
Post booster dose: 14 days, GMs (95% CI)	2158	2047	0.55
	(1897–2455)	(1798–2330)	

## Data Availability

The data supporting this study’s findings are available from the corresponding author upon reasonable request.

## Supplementary Materials

The following are available online at https://www.mdpi.com/article/10.3390/vaccines10101646/s1, Table S1: Baseline characteristics., Table S2: Solicited reactogenicity during 7 days after BNT162b2 primary series vaccination in healthy adolescents by vaccination groups., Table S3: Spike-specific T cell response and RBD-specific memory B cell response after BNT162b2 primary series vaccination in healthy adolescents by vaccination groups.
