# Supplementary material for: Immunogenicity of a Fractional Dose of mRNA BNT162b2 COVID-19 Vaccine for Primary Series and Booster Vaccination among Healthy Adolescents
Source: Vaccines (Basel). 2022 Sep 30;10(10):1646. doi: 10.3390/vaccines10101646 (PMC9609361; doi:10.3390/vaccines10101646)
Supplement: Supplementary file 1 [file vaccines-10-01646-s001.zip › vaccines-1924205-supplementary.pdf]

Table S1: Baseline characteristics.

	3-week interval			6-week interval		
	**3wPZ30/30**	**3wPZ30/20**	**3wPZ20/20**	**6wPZ30/30**	**6wPZ30/20**	**6wPZ20/20**
	**n=20**	**n=20**	**n=19**	**n=20**	**n=19**	**n=20**
**Male, n (%)**	8 (40.0)	8 (40.0)	9 (47.4)	8 (42.1)	8 (42.1)	9 (45.0)
**Age (years), mean (SD)**	15.3 (1.6)	14.8 (1.7)	15 (1.6)	16.2 (1.5)	15.2 (1.6)	14.3 (1.7)

* 3wPZ30/30: 3-week interval of BNT162b2 30 µg/30 µg; 3wPZ30/20: 3-week interval of BNT162b2 30 µg/20 µg; 3wPZ20/20: 3-week interval of BNT162b2 20 µg/20 µg; 6wPZ30/30: 6-week interval of BNT162b2 30 µg/30 µg; 6wPZ30/20: 6-week interval of BNT162b2 30 µg/20 µg; 6wPZ20/20: 6-week interval of BNT162b2 20 µg/20 µg

Table S2: Solicited reactogenicity during 7 days after BNT162b2 primary series vaccination in healthy adolescents, by vaccination groups.

**Dose 1**	**PZ30/30 n=40**	**PZ30/20 n=39**	**PZ20/20 n=39**
**Pain**	**32 (80.0)**	**33 (84.6)**	**33 (84.6)**
Pain grading			
• *Grade 1*	22 (55.0)	25 (64.1)	23 (59.0)
• *Grade 2*	7 (17.5)	7 (17.9)	9 (23.0)
• *Grade 3*	3 (7.5)	1 (2.6)	1 (2.6)
**Chill**	**4 (10.0)**	**3 (7.7)**	**5 (12.8)**
Fever grading			
• *Grade 1*	1 (2.5)	2 (5.1)	3 (7.7)
• *Grade 2*	1 (2.5)	1 (2.6)	1 (2.6)
• *Grade 3*	2 (5.0)	0 (0)	1 (2.6)
**Headache**	**14 (35.0)**	**12 (30.1)**	**11(28.2)**
Headache grading			
• *Grade 1*	12 (30.0)	10 (25.6)	9 (23.0)
• *Grade 2*	1 (2.5)	2 (5.1)	1 (2.6)
• *Grade 3*	1 (2.5)	0 (0)	1 (2.6)
**Fatigue**	**18 (45.0)**	**13 (33.3)**	**13 (33.3)**
Fatigue grading			
• *Grade 1*	13 (32.5)	10 (25.6)	7 (17.9)
• *Grade 2*	2 (5.0)	2 (5.1)	5 (12.8)
• *Grade 3*	3 (7.5)	1 (2.6)	1 (2.6)
**Myalgia**	**17 (42.5)**	**8 (20.5)**	**14 (35.9)**
Myalgia grading			
• *Grade 1*	11 (27.5)	5 (12.8)	8 (20.5)
• *Grade 2*	3 (7.5)	3 (7.7)	5 (12.8)
• *Grade 3*	3 (7.5)	0 (0)	1 (2.6)

**Arthralgia**	**5 (12.5)**	**4 (10.3)**	**6 (15.4)**
Arthralgia grading			
• *Grade 1*	4 (10.0)	3 (7.7)	3 (7.7)
• *Grade 2*	0 (0)	1 (2.6)	2 (5.1)
• *Grade 3*	1 (2.5)	0 (0)	1 (2.6)
**Vomiting**			
• *Grade 1*	0 (0)	1 (2.6)	0 (0)
**Diarrhea**	3 (7.5)	3 (7.7)	2 (5.1)
• *Grade 1*	3 (7.5)	3 (7.7)	2 (5.1)
• *Grade 2*	0 (0)	0 (0)	0 (0)

Dose2	PZ30/30 n=39	PZ30/20 n=39	PZ20/20 n=39
**Pain**	**36 (92.3)**	**30 (76.9)**	**25 (64.1)**
Pain grading			
• *Grade 1*	18 (46.1)	16 (41.0)	13 (33.3)
• *Grade 2*	13 (33.3)	12 (30.7)	10 (25.6)
• *Grade 3*	5 (12.8)	2 (5.1)	2 (5.1)
**Swelling**	**3 (7.7)**	**3 (7.7)**	**0 (0)**
Swelling grading			
• *Grade 1*	3 (7.7)	3 (7.7)	0 (0)
• *Grade 2*	0 (0)	0 (0)	0 (0)
Redness			
• *Grade 1*	2 (5.1)	1 (2.6)	1 (2.6)
**Fever**	**4 (10.3)**	**2 (5.1)**	**3 (7.7)**
Fever grading			
• *Grade 1*	4 (10.3)	2 (5.1)	1 (2.6)
• *Grade 2*	0 (0)	0 (0)	1 (2.6)
• *Grade 3*	0 (0)	0 (0)	1 (2.6)
**Chills**	**14 (35.9)**	**7 (17.9)**	**3 (7.7)**
Chills grading			
• *Grade 1*	9 (23.0)	5 (12.8)	3 (7.7)
• *Grade 2*	5 (12.8)	1 (2.6)	0 (0)
• *Grade 3*	0 (0)	1 (2.6)	0 (0)
**Headache**	**18 (46.2)**	**13 (33.3)**	**9 (23.0)**
Headache grading			
• *Grade 1*	8 (20.5)	8 (20.5)	5 (12.8)
• *Grade 2*	9 (23.0)	5 (12.8)	4 (10.3)
• *Grade 3*	1 (2.6)	0 (0)	0 (0)
**Fatigue**	**21 (53.8)**	**14 (35.9)**	**11 (28.2)**
Fatigue grading			
• *Grade 1*	14 (35.9)	10 (25.6)	7 (17.9)
• *Grade 2*	6 (15.4)	3 (7.7)	4 (10.3)
• *Grade 3*	1 (2.6)	1 (2.6)	0 (0)
**Myalgia**	**20 (51.3)**	**10 (25.6)**	**13 (33.3)**
Myalgia grading			
• *Grade 1*	10 (25.6)	6 (15.4)	9 (23.0)
• *Grade 2*	7 (17.9)	4 (10.3)	4 (10.3)
• *Grade 3*	3 (7.7)	0 (0)	0 (0)
**Arthralgia**	**4 (10.3)**	**3 (7.7)**	**1 (2.6)**

Arthralgia grading			
• *Grade 1*	2 (5.1)	2 (5.1)	1 (2.6)
• *Grade 2*	2 (5.1)	1 (2.6)	0 (0)
**Vomiting**			
• *Grade 1*	1 (2.6)	0 (0)	1 (2.6)
**Diarrhea**	**1 (2.6)**	**6 (15.4)**	**1 (2.6)**
• *Grade 1*	1 (2.6)	5 (12.8)	1 (2.6)
• *Grade 3*	0 (0)	1 (2.6)	0 (0)

Table S3: Spike-specific T cell response and RBD-specific memory B cell response after BNT162b2 primary series vaccination in healthy adolescents by vaccination groups.

	**3-week interval**			**6-week interval**		
	**3wPZ30/30**	**3wPZ30/20**	**3wPZ20/20**	**6wPZ30/30**	**6wPZ30/20**	**6wPZ20/20**
**Spike-specific T cell response (SFU/10^6 PBMCs)**						
**Post 2nd dose: 14-21 days, median (IQR)**	n=10	n=10	n=9	n=11	n=9	n=9
	136 (116-220)	202 (148-292)	196 (108-352)	128 (60-264)	184 (88-268)	200 (132-288)
**Post 2nd dose: 5 months, median (IQR)**	n=10	n=8	n=8	n=2	n=5	n=6
	42 (12-52)	48 (24-96)	54 (34-72)	8 (0-16)	44 (32-64)	106 (32-132)
**RBD-specific memory B cell response (SFU/10^6 PBMCs)**						
**Post 2nd dose: 5 months, median (IQR)**	n=10	n=8	n=8	n=2	n=5	n=6
	33 (16-54)	22 (7-49)	37 (23-64)	9 (6-12)	0 (0-2)	6 (0-34)

*PBMC: Peripheral blood mononuclear cell; RBD: Receptor binding domain; SFU: Spot forming unit; 3wPZ30/30: 3-week interval of BNT162b2 30 µg/30 µg; 3wPZ30/20: 3-week interval of BNT162b2 30 µg/20 µg; 3wPZ20/20: 3-week interval of BNT162b2 20 µg/20 µg; 6wPZ30/30: 6-week interval of BNT162b2 30 µg/30 µg; 6wPZ30/20: 6-week interval of BNT162b2 30 µg/20 µg; 6wPZ20/20: 6-week interval of BNT162b2 20 µg/20 µg
